# Bioavailability of Trace Elements in Beans and Zinc-Biofortified Wheat in Pigs

**DOI:** 10.1007/s12011-012-9453-2

**Published:** 2012-05-26

**Authors:** Dorthe Carlson, Jan Værum Nørgaard, Bulent Torun, Ismail Cakmak, Hanne Damgaard Poulsen

**Affiliations:** 1 Department of Animal Science, Aarhus University, Foulum, P.O. Box 50, DK-8830 Tjele, Denmark; 2 Department of Soil Science and Plant Nutrition, Cukurova University, Adana, Turkey; 3 Faculty of Engineering and Natural Sciences, Sabanci University, 34956 Istanbul, Turkey

**Keywords:** Iron, Zinc, Bean, Wheat, Bioavailability, Pigs

## Abstract

The objectives of this experiment were to study bioavailability of trace elements in beans and wheat containing different levels of zinc and to study how the water solubility of trace elements was related to the bioavailability in pigs. Three wheat and two bean types were used: wheat of Danish origin as a control (CtrlW), two Turkish wheat types low (LZnW) and high (HZnW) in zinc, a common bean (Com), and a faba bean (Faba). Two diets were composed by combining 81 % CtrlW and 19 % Com or Faba beans. Solubility was measured as the trace element concentration in the supernatant of feedstuffs, and diets incubated in distilled water at pH 4 and 38°C for 3 h. The bioavailability of zinc and copper of the three wheat types and the two bean-containing diets were evaluated in the pigs by collection of urine and feces for 7 days. The solubility of zinc was 34–63 %, copper 18–42 %, and iron 3–11 %. The zinc apparent digestibility in pigs was similar in the three wheat groups (11–14 %), but was significantly higher in the CtrlW+Faba group (23 %) and negative in the CtrlW+Com group (−30 %). The apparent digestibility of copper was higher in the HZnW (27 %) and CtrlW+Faba (33 %) groups than in the CtrlW (17 %) and LZnW (18 %) groups. The apparent copper digestibility of the CtrlW+Com diet was negative (−7 %). The solubility and digestibility results did not reflect the concentration in feedstuffs. The in vitro results of water solubility showed no relationship to the results of trace mineral bioavailability in pigs.

## Introduction

Biofortification of food with micronutrients of special importance for health is recognized as a strategy to alleviate the consequences of malnutrition at a relatively low cost [1] which is of high significance in developing countries. Zinc and iron deficiency is widespread in low-income countries because of a low consumption of animal products with a high content of bioavailable dietary iron and zinc. Instead, in these countries, it is common to have a high consumption of cereal grains and legumes which contain inhibitors of zinc absorption [2, 3].

Great progress has been made during the last decade in order to develop cereal and legume lines with a high content of zinc and iron [4, 5]. Potentially, this effort is expected to improve the zinc and iron status of humans in developing countries. In order to improve the human zinc and iron status, it is essential that the trace elements from these dietary sources are available for absorption. Bioavailability of trace elements is, in this experiment, therefore defined as the proportion of net absorbed nutrients in relation to ingested nutrients, i.e., the apparent digestibility.

The bioavailability of trace elements from cereals and legumes depends on different chemical characteristics. One factor of special importance is the content of phytate which can be present in high concentrations in cereal grains and legumes. Phytate complexes bind divalent cations resulting in inhibition of trace element absorption [6]. It is expected that a mineral is available for absorption in the small intestine when it is on ionic form and thereby is soluble [7].

Different approaches can be used to evaluate zinc-fortified cereals and legumes as dietary zinc sources for humans. The most appropriate way is to evaluate the different lines directly in human studies. However, these studies are expensive and very time-consuming. An alternative way is to perform bioavailability studies on animals, and the pig is known to be a very good model for humans [8, 9]. Alternatively or as a supplement, in vitro screening may be a quick method to study mineral bioavailability in cereals and legumes. Throughout the world, several in vitro methods have already been developed for this purpose [10–12]. Common for these methods are that they simulate gastrointestinal digestion and physiology and thereby build on rather complicated assays. It is therefore relevant to study if bioavailability can be assessed by a more straightforward in vitro method. The hypothesis was that assessment of the water solubility of zinc and iron from cereals and legumes can be used to rank food in respect of zinc and iron bioavailability in humans.

Consequently, the objectives of the study were to test a simple in vitro method to estimate zinc and iron solubility in different wheat and bean lines, and to evaluate zinc and iron bioavailability of the same wheat and bean lines in pigs. Hence, the overall aim was to validate the in vitro assay as a screening method for ranking wheat and bean lines in terms of their potential as sources of dietary zinc and iron.

## Materials and Methods

### Feed

Three varieties of wheat and two varieties of beans varying in zinc content were produced. Two wheat batches from Denmark (*Triticum aestivum*, L. cv. Deben) and Turkey (*T. aestivum*, L. cv. Adana 99) were produced on fields in Denmark with no specific zinc fertilization. Zinc fertilization of wheat in Turkey has been realized by spraying 0.5 % ZnSO_4_ to foliar at booting and early milk stages as described by Cakmak et al. [13]. Foliar spray of Zn to wheat in Turkey did not cause any significant change in grain yield, but increased grain Zn content from 23 mg/kg (control; no Zn spray) to 43 mg/kg. Faba beans (*Vicia faba)* were produced in Denmark under organic conditions. The common Andean type bean (*Phaseolus vulgaris* L. var. NUA) variety was grown in East Africa.

The diets fed to the pigs consisted of wheat as the only component or a combination of 19 % bean and 81 % Danish wheat. Thus, the zinc and iron bioavailability of wheat was determined by a direct method and those of beans by an indirect method. The indirect method is often used to evaluate digestibility of feedstuffs which cannot be fed in great amounts due to e.g. a high content of antinutritional factors or low palatability [14]. Diets were processed on a hammer mill to a maximum particle size of 3 mm. The chemical composition of the feed ingredients is shown in Table 1.

**Table 1:** Dietary ingredients and analyzed chemical composition of the diet fed to all experimental pigs in the preparation period (as-fed basis)

Ingredient	%
Barley	51.4
Wheat	20.0
Animal fat	2.00
Soybean meal, toasted	23.0
Sugar beet molasses	1.00
l-lysine HCl (78 %)	0.08
DL-methionine (99 %)	0.02
Monocalcium phosphate	0.48
Calcium carbonate	1.30
Salt	0.33
Mineral and vitamin mixture^a^	0.40
Phytase (Natuphos 5000)	0.02

^a^Provided the following quantities of vitamins and minerals per kilogram of complete diet: 15,000 IU of vitamin A, 2,000 IU of vitamin D_3_, 91 mg of α-tocopherol, 1.5 mg of menaphthone,1 mg of thiamin, 6 mg of riboflavin, 20 mg of d-pantothenic acid, 25 mg of niacin, 0.025 mg of biotin, 0.025 mg of cyanocobalamin, 1.5 mg of pyridoxine, 160 mg of Cu as CuSO_4_^.^5H_2_O, 40 mg of Mn as MnO, 1 mg I as Ca(IO_3_)_2_, and 0.4 Se as Na_2_SeO_3_

The wheat of Danish origin was considered as a control and designated as CtrlW. The wheat without Zn spray and lower grain Zn was designated as LZnW and the wheat with high Zn as HZnW. The common bean was designated as Com and the faba bean as Faba.

### In Vitro Study

The in vitro study was performed on the three batches of wheat (CtrlW, LZnW, and HZnW), the two batches of bean (Com and Faba), and on two mixed CtrlW and Com or Faba bean diets (CtrlW+Com and CtrlW+Faba). For in vitro studies, the feed ingredients and mixtures were milled on a 2-mm sieve.

All equipment were washed in acid and rinsed with deionized water before use to avoid zinc and iron contamination. The assay involved 20 g of feed mixed with 80 g of redistilled water in 100 ml conique balloons. These mixtures were incubated and continuously stirred on a magnetic stirrer in a water bath at 38°C. Hydrochloric acid (0.5 M) was added whenever necessary to maintain the pH at 4, which is within the range often seen in the stomach of pigs [15, 16]. The mixtures were incubated under these conditions for 3 h and then immediately placed on ice before the samples were centrifuged at 11,000×*g* for 30 min. The supernatant was removed and stored at −20°C until chemical analyses. The procedure was repeated twice for each feed ingredient and mixture.

### In Vivo Study

The experiment comprised eight litters of five female crossbred (Landrace × Yorkshire × Duroc) pigs and started when the live weight was approximately 35 kg. During a 2-week preparation period, the animals were fed a regular grower diet based on barley, wheat, and soybean meal supplemented with lysine, methionine, vitamins, and minerals to fulfill the pigs’ requirement for all nutrients except zinc and iron (Table 1). During the 2-week preparation period, the pigs were placed in individual pens and fed ad libitum. After the preparation period, at a body weight of 43.3 (±2.2) kg, the pigs were assigned according to litter to one of five dietary treatments also used in the in vitro study: CtrlW, LZnW, HZnW, CtrlW+Com, and CtrlW+Faba.

The pigs were placed in stainless steel cages for a 5-day adaptation period followed by a 7-day collection period. The body weight of the animals was recorded at the beginning and at the end of the balance period. During the experimental period, the pigs were fed 1,400 g/day, and feed residuals were registered twice daily. Throughout the adaptation and balance period, the pigs were fed twice daily at 0745 and at 1430 hours, respectively. The pigs had continuous access to demineralized water. Catheters were inserted into the urine bladder to separate urine and feces, and feces were collected by adhesion of plastic bags to the back of the pigs. Furthermore, special care was taken to avoid contamination (stainless steel equipment, acid washing of equipment and utensils). Urine and faces were collected and weighed during the balance period.

### Chemical Analyses

All analyses were performed in duplicate. The samples of wheat, beans, diets, and feces were analyzed for dry matter (DM), ash, zinc, iron, copper, calcium, phosphorus, and protein. Wheat and beans were analyzed for amino acids, phytic acid, and phytase activity. The DM content was determined by oven-drying at 103°C for 20 h. The samples were reduced to ashes at 450°C, and the ash was digested in a 21.7 % nitric acid solution for mineral analyses. The supernatant from the in vitro studies and the urine samples were reduced to ashes in nitric acid (14.4 M) and perchloric acid (12 M) at 200°C. The concentrations of zinc, copper, iron, and calcium were determined by atomic absorption spectrophotometry (S Series, Thermo Electron Cooperation, Bremen, Germany), and with regard to the urine samples, zinc, copper, and iron were determined by ICP-MS (X series^II^, Thermo Electron Corporation, Bremen, Germany). Phosphorus was analyzed by the colorimetric vanadomolybdate procedure [17]. Phytate P was measured according to Haug and Lantzsch [18]. Phytase activity was determined by the method of Engelen et al. [19] where one phytase unit (FTU) defines the amount of enzyme which liberates 1 mmol inorganic orthophosphate per minute from 0.0051 mol/l sodium phytate at pH 5.5 and 37°C.

The nitrogen content in feedstuffs, diets, and feces was analyzed by the Dumas method [20] and in urine by a modified Kjeldahl method [21], and the crude protein content in feedstuffs and diets was quantified as total nitrogen × 6.25. Amino acids in diets were hydrolyzed for 23 h at 110°C with or without performic acid oxidation, and amino acids were separated by ion exchange chromatography and quantified by photometric detection after ninhydrin reaction [22].

### Calculations and Statistics

#### In Vitro Data

The solubility of trace elements (TE) was calculated from the trace element content of the dietary ingredients and the trace element content of the supernatant after incubation in vitro.

TE solubility (percentage) = TE in 80 ml of supernatant (milligrams)/TE in 20 g feed (milligrams) × 100

Soluble TE (milligrams per kilogram of feed) = TE solubility (percentage) × TE in feed (milligrams per kilogram DM)

The results from the in vitro study are presented as the mean of two measurements ± standard deviations.

#### In Vivo Data

Statistical analysis was performed by use of generalized linear models in SAS [23]. The effect of dietary treatment on zinc and copper excretion in urine and faces, net absorption, and net absorption relative to intake together with the net retention was analyzed in a model with litter as the blocking factor. The same model was used to analyze the effect of diet on the DM and N digestibility. Furthermore, when there was a significance level of *P* < 0.05, the least square estimates were compared using the PDIFF option [23]. The results are presented as the least squares means and the standard error of mean (SEM).

## Results

The chemical composition of the dietary ingredients is presented in Table 2. It is clear that the two Turkish wheat samples differed in zinc content (23.3 and 42.8 mg/kg DM, respectively) and slightly in iron content (38.2 and 41.7 mg/kg DM, respectively), whereas the content of all other nutrients was almost identical in the two Turkish wheat samples. Compared to the Turkish wheat samples, the Danish wheat was lower in protein (126 vs. 147 and 148 g/kg DM, respectively), lower in all the analyzed amino acids, in phytate P (2.0 vs. 3.0 g/kg DM), and in all minerals except Ca which was similar for all wheat types (0.5 g/kg DM). The phytase activity was slightly higher in the Danish wheat (949 FTU/kg DM) compared to the Turkish wheat (817–855 FTU/kg DM).

**Table 2:** Chemical composition of the control wheat (CtrlW), wheat low (LZnW) or high (HZnW) in zinc, common (Com) or faba (Faba) beans used in the in vitro and in vivo studies

	CtrlW	LZnW	HZnW	Com	Faba
Dry matter (DM) (%)	88.7	89.2	87.7	87.1	85.3
Crude ash (g/kg DM)	15	18	19	44	39
Crude protein (Nx6.25) (g/kg DM)	126	147	148	248	309
Lysine (g/kg DM)	3.3	3.9	3.8	17.0	20.0
Methionine (g/kg DM)	1.9	2.3	2.3	2.7	1.9
Cysteine (g/kg DM)	2.7	3.2	3.3	3.0	2.9
Histidine (g/kg DM)	2.8	3.4	3.4	7.6	8.1
Threonine (g/kg DM)	3.5	4.2	4.2	10.8	10.8
Phytate P (g/kg DM)	2.0	3.0	3.0	2.5	2.2
Phytase activity (FTU/kg DM)	949	817	855	10	n.d.
P (g/kg DM)	2.8	3.9	3.9	5.2	6.3
Ca (g/kg DM)	0.5	0.5	0.5	1.8	1.8
Zn (mg/kg DM)	15.6	23.3	42.8	29.9	41.4
Cu (mg/kg DM)	3.5	4.2	4.8	10.4	10.0
Fe (mg/kg DM)	34.2	38.2	41.7	78.9	44.0

*n.d.* not detectable

The two bean types also differed in the zinc and iron content with the lowest zinc content in the Com beans (29.9 mg/kg DM) compared to the Faba beans (41.4 mg/kg DM). In contrast, the highest iron content was in the Com beans (78.9 mg/kg DM) compared to the Faba beans (44.0 mg/kg DM). Furthermore, the bean types differed in the protein content with the highest protein level in the Faba beans (248 vs. 309 g/kg DM), and the amino acid profile of the two bean types also varied notably (Table 2).

The analyzed and calculated mineral content of the two wheat and bean mixtures (CtrlW+Com and CtrlW+Faba) is shown in Table 3. For P, Ca, Zn, and Cu, the analyzed and calculated mineral content was almost identical. However, there was a large discrepancy between the analyzed and calculated concentration of iron indicating that pollution with iron took place during the milling process. Consequently, the in vitro as well as in vivo results on iron based on the contaminated diets are considered irrelevant and are not presented (Tables 4 and 5).

**Table 3:** Mineral content in dry matter (DM) of mixtures of 81 % wheat and 19 % common (CtrlW+Com) or faba (CtrlW+Faba) beans used in the in vitro and in vivo studies

	CtrlW+Com		CtrlW+Faba	
	Analyzed	Calculated	Analyzed	Calculated
P (g/kg DM)	3.2	3.3	3.4	3.5
Ca (g/kg DM)	0.7	0.9	0.7	0.8
Zn (mg/kg DM)	19.4	18.3	20.5	20.5
Cu (mg/kg DM)	4.8	4.8	4.8	4.7
Fe (mg/kg DM)	97.0	42.7	79.5	36.1

The calculated concentrations are based on the content measured in the individual ingredients as shown in Table 2

**Table 4:** In vitro solubility at pH 4 of trace minerals in control wheat (CtrlW), wheat low (LZnW) or high (HZnW) in zinc, common (Com) or faba (Faba) beans and mixtures of the control wheat (81 %) and common beans (CtrlW+Com) (19 %) or faba beans (Ctrl+Faba) (19 %)

	CtrlW	LZnW	HZnW	Com	Faba	CtrlW+Com	CtrlW+Faba
Solubility (%)							
Zn	63.3 ± 2.0	55 ± 1.0	50.8 ± 0.9	50 ± 5.2	34.4 ± 0.2	55.6 ± 0.1	51.1 ± 0.7
Cu	19.0 ± 0.2	23.5 ± 1.9	18.4 ± 0.1	42.1 ± 0.1	28.0 ± 0.7	22.5 ± 1.8	22.8 ± 0.5
Fe	5.9 ± 0.2	11.2 ± 3.7	7.1 ± 0	8.7 ± 0.2	3.8 ± 0.1	n.a.	n.a.
Soluble content (mg/kg DM)							
Zn	9.9 ±0.3	12.8 ± 0.2	21.7 ± 0.4	14.9 ± 0	14.3 ± 0.1	10.8 ± 0	10.5 ± 0.1
Cu	0.7	1.0 ± 0.1	0.9	4.4	2.8 ± 0.1	1.1 ± 0.1	1.1
Fe	2.0 ± 0.1	4.3 ± 1.4	3.0	6.8 ± 0.1	1.7	n.a.	n.a.

The results are presented as the mean of two measurements ± standard deviations

*n.a.* not analyzed due to contamination

**Table 5:** Dry matter (DM) and nitrogen (N) digestibility, daily zinc (Zn) and copper (Cu) intake, excretion, net absorption, apparent digestibility, and retention in pigs fed control wheat (CtrlW), wheat low (LZnW) or high (HZnW) in zinc, or mixtures of control wheat and common (Com) or faba (Faba) beans

	CtrlW	LZnW	HZnW	CtrlW+Com	CtrlW+Faba	SEM	*P* value
Number of pigs (*n*)	8	8	8	5	8		
DM digest. (%)	91ab	89b	89b	64c	93a	3.1	<0.001
N intake (g)	23.9c	28.2b	28.4b	9.5d	32.6a	1.3	<0.001
N in feces (g)	3.8a	4.4a	4.4a	2.9b	3.9a	0.6	0.002
N digest. (%)	84b	85b	85b	39c	95a	3.9	<0.001
N in urine (g)	14.9b	19.2ab	21.5a	8.1c	13.9bc	5.4	0.002
N reten. (g)	5.2b	5.2b	2.5bc	−1.5c	14.8a	5.7	<0.001
Zn intake (mg)	19.5d	28.7b	57.5a	7.3e	25.2c	1.4	<0.001
Zn in feces (mg)	16.6c	24.6b	50.8a	9.4d	19.5c	3.5	<0.001
Zn digest. (%)	14.3b	14.4b	11.7b	−29.9c	22.8a	7.1	<0.001
Zn in urine (mg)	0.4	0.6	0.6	0.3	0.5	0.2	0.27
Zn reten. (mg)	2.4b	3.6ab	6.2a	−2.4c	5.3ab	3.3	0.002
Cu intake (mg)	4.2c	5.5b	5.8a	1.9d	5.9a	0.23	<0.001
Cu in feces (mg)	3.5c	4.5a	4.2ab	2.0d	4.0b	0.42	<0.001
Cu digest. (%)	17b	18b	27a	−7c	33a	6.7	<0.001
Cu in urine (mg)	0.03b	0.04b	0.04b	0.02c	0.06a	0.01	<0.001
Cu reten. (mg)	0.7c	1.0c	1.5b	−0.1d	1.9a	0.4	<0.001

Means within rows without a common lowercase letter differ (*P* ≤ 0.05)

The in vitro results are presented in Table 4. It shows that 34–63 % of the zinc, 18–42 % of the copper, and 3–11 % of the iron content in wheat and beans were soluble at pH 4 and 20°C after 3 h. When comparing the three wheat types, it is clear that the control wheat had the highest zinc solubility, whereas the LZnW had the highest copper and iron solubility. When comparing the two bean types, it was found that the Com beans had the highest solubility of the three trace elements when measured in percent of total content of the individual trace elements.

The soluble zinc content of the three wheat samples was 9.9, 12.8, and 21.7 mg/kg DM in the CtrlW, LZnW, and HZnW, respectively. The LZnW had the highest content of soluble copper and iron, and the control wheat had the lowest content of soluble copper and iron when given as milligrams per kilogram DM. In the CtrlW+Com group, three pigs were taken out of the experiment due to very low feed intake, and consequently, these pigs were considered as outliers, and their data were excluded from the data set.

In spite of the fact that all pigs were offered 1,400 g feed per day, irrespective of the dietary treatment, the average daily feed intake was 1,336, 1,380, 1,370, 444, and 1,400 g/day in the CtrlW, LZnW, HZnW, CtrlW+Com, and CtrlW+Faba diets, respectively. Consequently, the CtrlW+Com group consumed less (*P* < 0.001) feed compared to the other groups. The average weight gains of the experimental pigs in the balance period were 165, 190, 102, −251, and 386 g/day for the control, LZnW, HZnW, CtrlW+Com, and CtrlW+Faba groups, respectively.

The apparent digestibility of DM was 64 % for the CtrlW+Com diet which was lower (*P* < 0.001) than for the other groups where the DM digestibility was around 90 % as seen under normal feed conditions. This emphasizes that the results obtained in pigs fed the CtrlW+Com diet may be dubious. The apparent digestibility of N was also reduced (*P* < 0.05) in the group fed the CtrlW+Com diet, and this resulted in a negative N retention (*P* < 0.05). The greatest (*P* < 0.05) N retention was found for the group fed the CtrlW+Faba diet.

The daily zinc intake differed between all dietary groups (*P* < 0.001) with the highest intake in pigs fed the HZnW (57.5 mg/day) and the lowest zinc intake in pigs fed the CtrlW+Com diet (7.3 mg/day; Table 5). Consequently, the net zinc absorption in milligrams per day differed (*P* < 0.001) between the dietary groups with the highest zinc absorption in the HZnW group (6.8 mg/day). The low zinc intake of pigs fed CtrlW+Com resulted in a net excretion of 2.0 mg zinc/day. The digestibility of zinc was similar for the three wheat groups (11–14 %), but was significantly higher in the CtrlW+Faba group (22.8 %) and negative in the CtrlW+Com group (−29.9 %). The zinc excretion in urine was very low (below 1 mg/day) for all treatments.

The daily copper intake also differed (*P* < 0.001) between the dietary groups with the highest intake in the HZnW and CtrlW+Faba groups (5.8 and 5.9 mg/day, respectively) and the lowest intake in the CtrlW+Com group (1.9 mg/day). The apparent copper digestibility was higher in the HZnW (27 %) and CtrlW+Faba (33 %) groups than in the CtrlW (17 %) and LZnW (18 %) groups. The lowest apparent copper digestibility was in the CtrlW+Com (−7 %) group. The copper excretion in urine was very low but was affected (*P* < 0.001) by the intake, and hence, the highest excretion was in the CtrlW+Faba group (0.06 mg/day) and the lowest excretion in the CtrlW+Com group (0.02 mg/day).

## Discussion

The in vitro study indicated that at pH 4, which is typically seen in gastric contents in pigs fed cereal-based diets [15, 16], the solubility of zinc was higher than the solubility of copper which was higher than the solubility of iron after 3 h of incubation. This order of solubility was the same in both wheat and beans. Phytate strongly binds positively charged divalent ions, and the decreasing order of stability of mineral phytate complexes in vitro is Zn^2+^, Cu^2+^, Ni^2+^, Co^2+^, Mn^2+^, Ca^2+^, and Fe^2+^ [24]. Hence, the reduced order of solubility of zinc, copper, and iron found in the current in vitro study was completely different and therefore cannot be related to the affinity of phytate for the individual trace elements.

The difference in solubility among trace elements may partly be explained by the localization of the trace elements in the grains. In wheat, the highest concentrations of iron and zinc are present in the aleurone and the embryo, but with some presence of zinc in the endosperm [5, 13]. It may be that a higher presence of zinc in the starchy endosperm will result in a higher solubility of zinc compared to iron under the in vitro conditions in the present study.

In the Faba-fed group, the total zinc intake of pigs was lower in the CtrlW and the LZnW groups, and when the bioavailability of zinc from the faba beans per se was calculated by the difference method, the absorption of zinc and copper from faba beans was 39 and 56 %, respectively (data not shown). Due to the very low zinc intake in the Com bean group, it was not possible to calculate a reliable value for the bioavailability of zinc from Com beans by the difference method. The observed zinc bioavailability in the present study around 20 % agrees with former results in pigs fed a barley-, wheat-, and soybean-based diet [25]. These authors also found the urinary zinc excretion to be less than 1 mg/day corresponding to the obligatory endogenous zinc loss.

The amount of protein has a positive effect on the zinc absorption. Amino acids, such as histidine and methionine, and other low molecular weight ions are known to have a positive effect on the zinc absorption [26]. Faba beans are high in histidine compared to the studied wheat lines. Histidine is a good chelator of zinc, and clinical studies on humans have shown a positive effect of histidine on the zinc absorption [26]. It would be of interest to study if the histidine content could explain the higher bioavailability of zinc in faba beans.

**Fig. 1:**
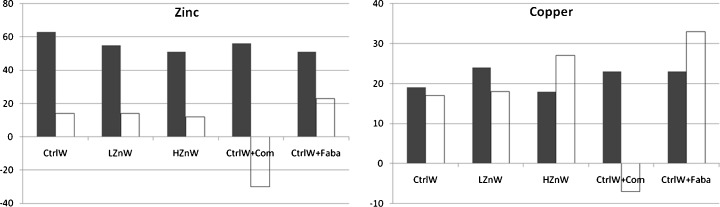
Comparison of zinc and copper solubility (percentage) (*solid bars*) and apparent digestibility (percentage) in pigs (*open bars*) of control wheat (*CtrlW*), wheat low (*LZnW*) or high (*HZnW*) in zinc, or mixtures of control wheat and common (*Com*) or faba (*Faba*) beans

The present study showed quite reasonable similarities in results of the copper solubility and bioavailability measured in vitro and in vivo, respectively, for all diets except the diet including common beans (Fig. 1). However concerning zinc, the solubility results were two- to fivefold higher compared to the bioavailability results. This may be due to the 3-h incubation time, which may have been too long because former pig studies have shown that about 25 and 40 % of the DM intake already have left the stomach 1 and 2 h after eating, respectively [16]. The in vitro assay used in the present study was developed to be used as a very simple screening method for ranking wheat and bean lines in terms of their potential as sources of dietary zinc. However, the applied in vitro assay needs further development in e.g. incubation time to imitate the digestive processes and retention time in the gastrointestinal tract to be used in future zinc studies.

## Conclusions

The three types of wheat differed in their content of zinc. Zinc-fortified wheat had almost threefold the zinc content as the wheat type with the lowest content. The solubility of zinc, copper, and iron measured after 3 h of incubation at pH 4 and 38°C was the highest for zinc and the lowest for iron, and there was no indication that the solubility was directly dependent on the trace element concentration. Results of zinc and copper digestibility in pigs also did not reflect the content of the feedstuffs. The in vitro solubility showed similarities to the copper but not of zinc digestibility, indicating that the assay has to be further developed in order to simulate trace mineral bioavailability.
